# Angular assessment of joints in juvenile idiopathic arthritis

**DOI:** 10.1515/rir-2025-0001

**Published:** 2025-04-02

**Authors:** Sudip Banerjee, Atanu Adak, Debadyuti Dutta, Partha Pratim Pan, Manab Nandy, Avijit Hazra, Rakesh K Mondal

**Affiliations:** Department of Pediatrics, North Bengal Medical College, Darjeeling, India; Multidisciplinary Research Unit, Medical College Kolkata, Kolkata India; Department of Physical Medicine, North Bengal Medical College Darjeeling, Darjeeling India; Department of Pharmacology, Medical College Kolkata, Kolkata India; Department of Pharmacology, IPGMER and SSKM Hospital, Kolkata India; Department of Pediatrics, Medical College and Hospital, Kolkata India

**Keywords:** juvenile idiopathic arthritis, Joint deformities, goniometry, angular deviation

## Abstract

**Background:**

Joint deformities in juvenile idiopathic arthritis (JIA) are most common in children, are not defined in term of angular measurements. The study was aimed to evaluate the joint deformities in angular deviation of the afected joints in JIA patients.

**Methods:**

This cross-sectional study was conducted at Pediatric Rheumatology Clinic, North Bengal Medical College, West Bengal. The children aged 2–16 years diagnosed with JIA according to the International League of Associations for Rheumatology (ILAR) criteria were included in the study. Patients with co-morbid disease, hemodynamic instability, and other acute conditions were excluded. Angular measurements were performed using goniometer.

**Results:**

The mean age of children was (8.05 ± 3.20) years of which 57.5% was male and the disease duration associated with the deformities in JIA. The prevalent subtypes of JIA were Oligoarticular JIA (oligoJIA)(40%), followed by polyarticular JIA (pJIA) (35%) and systemic-onset JIA (sJIA) (12.5%). The commonly involved joint were knee (40%), followed by small joint of hand (32.5%), ankle (30%), wrist and foot (17.5% each), elbow (12.5%) and cervical joint (7.5%). In pJIA, duration of disease significantly (*P* = 0.017) associated with the number of affected joints. Mostly, wrist, knee and ankle deformities were observed in oligoJIA, pJIA and sJIA. The angular deviation (mean ± SD) of right and left knee were (2° ± 4.16°) and (1.87° ± 5.12°) in oligoJIA, (13.36° ± 17.03°) and (12.5° ± 15.08°) in pJIA and (3° ± 6.71°) and (2.4° ± 5.37°) in sJIA. Right ankle angular deviation were (2.62° ± 5.06), (5.43° ± 8.21°) and 4° ± 8.94° respectively in oligoJIA, pJIA and sJIA. The angular deviation of right and left wrist were (1.25° ± 3.41°) and (0.94° ± 3.75°) in oligoJIA, (4.07° ± 8.93°) and (4.14° ± 9.36°) in pJIA and (2.45° ± 5.37°) and (2° ± 4.47°) in sJIA.

**Conclusion:**

This study is the first study from India to quantify the angular deviation of deformed joints in JIA. Angular deviation could serve as a valuable parameter for monitoring disease progression across various JIA subtypes.

## Introduction

Juvenile idiopathic arthritis (JIA) is a chronic autoimmune disease that affects the children.^[1]^ The prevalence of JIA varies widely across different populations. Globally, the prevalence of JIA has been reported to range from 7 to 401 cases per 100, 000 children. In India, the prevalence of JIA is lower than the global average, with an estimated rate of 48 cases per 100, 000 children.^[2]^ In JIA, common skeletal abnormalities include joint deformity, discrepancy in limb length, valgus deformity of the knees and delay in growth, micrognathia, retrognathia, facial asymmetry, malocclusion (if temporomandibular joint involvement).^[3,4]^ Decreased joint motion in inflamed joints can leads to deformities like Boutonniere deformity, swan neck deformity, Z-deformities of thumb, spindling of fingers, subluxations, deviations, fixed flexion deformities, valgus and varus deformities in various joints.^[2,5,6]^ A few studies were performed on radiographic images of afected joints to monitor the JIA.^[7,8]^ However, joint involvement is classically described to define deformities in available literature, it has not defined with exact angular measurement in degree. There is a clear lack of studies regarding specific measurement of joint movement in JIA patients. Hence, the present study was designed to add a new measurable parameter for monitoring disease which may be useful for clinical practice. In this context, the angular deviation of joints was evaluated in various sub-categories of JIA using a goniometer.

## Materials and Methods

### Study Design

This cross-sectional study was conducted on JIA patients who visited the Pediatric Rheumatology Clinic and Physical Medicine Department at North Bengal Medical College in West Bengal, India.

### Patient Recruitment

A total of 40 children aged 2–16 years were recruited in the study both from indoor and outdoor of Pediatric Rheumatology Clinic and Physical medicine. Children with comorbid diseases, hemodynamic instability, congenital musculoskeletal anomalies, or joint deformities caused by other factors were excluded from the study. Newly diagnosed JIA patients were classified according to the International League of Associations for Rheumatology (ILAR) criteria. Patients were treated using standard monitoring and treatment approaches.

### Data Collection

Institutional ethical clearance (IEC/NBMC/2018–19/40) and consent from parents/caregivers/Legal guardians were taken prior to the study. Clinical details and exact angular measurement of affected joints were measured in degree using a Goniometer (Therapy-plus^TM^ 824, India) and recorded on predesigned proforma. Angle deviations were measured by goniometer with a range of 1º to180º. Detailed joint angle assessments were conducted as follows: Patients were positioned supine with their head and eyes directed upwards, upper limbs at their sides with elbows extended and palms facing upwards, hips and knees extended, and feet perpendicular to the horizontal plane. In this position, joint angles were measured with a goniometer placed laterally to the joints. The data was collected from May 2019 to April 2020.

### Statistical Analysis

Descriptive analysis was done in the form of proportion for categorical variables, mean or median for continuous variables. Data were checked for normality using tests for normality. Nonparametric tests were performed for variables that were not normally distributed. Differences between proportions were analyzed using the Chi-square test, and Spearman’s rank correlation coefficient (rho) was used to assess the correlation between variables. A *P*-value of less than 0.05 was considered statistically significant. All the data were analyzed using the SPSS (IBM Corp., Armonk, N.Y., USA) version 20.

## Results

**Figure 1:**
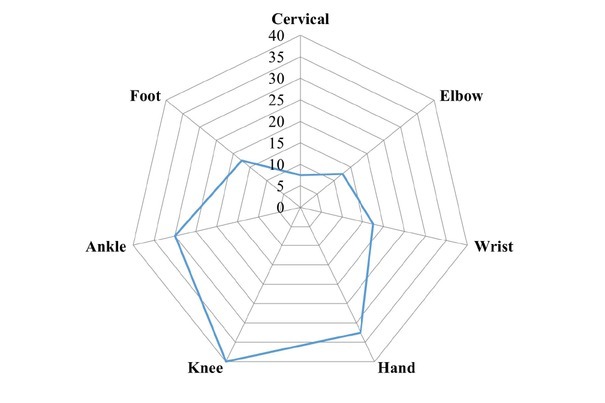
Distribution joint involvement of study subjects in juvenile idiopathic arthritis (JIA).

**Figure 2:**
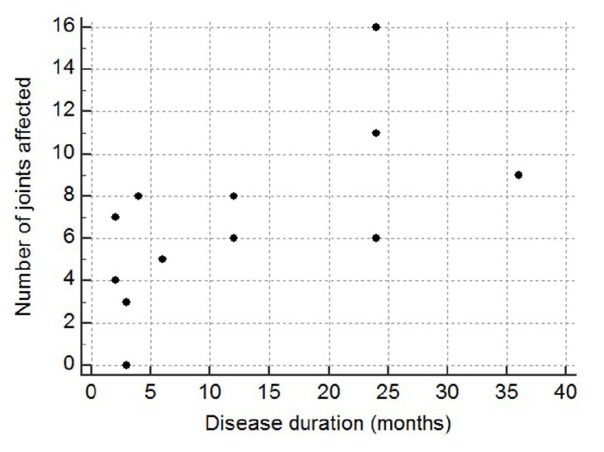
Spearman’s coefficient of rank correlation between disease duration and no. of joints affected in pJIA. Association was statistically significant where Spearman’s coefficient of rank correlation (rho) is 0.623, P = 0.017.

A total of 63 patients were screened and 40 patients with JIA were recruited for the assessment of joint deformities. The demographic data of the patients depicted in Table 1. The average age of the patients was 8.05 ± 3.2 years, of which majority was within the age group of 5–10 years (67.5%) followed by 11–15 years (15%) and < 5 years (12.5 %). JIA was more prevalent in males (57.5%) than females. The patients (57.5%) were diagnosed with JIA within 12 months of disease onset, 37.5% within 12–36 months, and 5% diagnosed more than 36 months after disease onset. The median (IQR) duration of disease was 6 months. Joint deformities were found in 72.5% patients, while 27.5% of were reported no deformities. The most prevalent subtype of JIA in the present study was Oligoarticular JIA (oligoJIA) (40%), followed by Polyarticular JIA (pJIA)(35%) and systemic-onset JIA (sJIA)(12.5%). The angular deviation of the other JIA subtypes were measured but not presented in the result. Most commonly involved joints were knee (40%), followed by small joint of hand (32.5%), ankle (30%), wrist and foot (17.5% each), elbow (12.5%) and cervical joint (7.5%)(Figure 1). Majority of the patients had oligoJIA (40%), followed by pJIA (35%) and sJIA (12.5%). Maximum involvement of joints was observed in pJIA and had a positive correlation (Spearman’s coefficient of rank correlation [rho]= 0.623, *P* = 0.017) with duration of the disease (Figure 2). Commonly, knee, right ankle and wrist deformities were observed in oligoJIA, pJIA and sJIA. The angular measurements and of different joint and their prevalence are depicted in Table 2. The angular deviation of right and left knee were 2° ± 4.16° and 1.87° ± 5.12° in oligoJIA, 13.36° ± 17.03° and 12.5° ± 15.08° in pJIA and 3° ± 6.71° and 2.4° ± 5.37° in sJIA. The prevalence of deformities of right and left knee were 25% and 18.75% in oligoJIA, 57.14% and 64.29% in pJIA and 20% in both joints of sJIA. Right ankle angular deviations in oligoJIA, pJIA, and sJIA were found to be 2.62° ± 5.06°, 5.43° ± 8.21°, and 4° ± 8.94°, respectively, with a prevalence of 25%, 42.86%, and 20%. Left ankle angular deviation were observed only in oligoJIA, pJIA, with deviation of 0.94° ± 2.72°and 5.29° ± 8.26°, respectively and a prevalence of 12.5% and 35.71%. The angular deviation of right and left wrist were 1.25° ± 3.41° and 0.94° ± 3.75° in oligo-JIA, 4.07° ± 8.93° and 4.14° ± 9.36°in pJIA and 2.45° ± 5.37° and 2° ± 4.47° in sJIA. The prevalence of right and left wrist angular deviation was 12.5% and 6.25% in oligoJIA, 21.43% in both wrists of pJIA, and 20% in both wrists of sJIA. Elbow, metatarsophalangeal (MTP), and proximal interphalangeal (PIP) joints were deviated only in oligoJIA and pJIA. In oligo-JIA, the angular deviations of the right and left elbows were 0.094° ± 2.72° and 5.00° ± 2.00°, respectively, with a prevalence of 12.5% and 6.25%. In pJIA, the angular deviations of the right and left elbows were 7.14° ± 13.26° and 5.64° ± 9.92°, respectively, with a prevalence of 28.57% in both. The angular deviations of the right and left MTP joints were 0.94° ± 3.75° and 0.62° ± 2.50° in oligoJIA, and 2.50° ± 1.00° and 2.43° ± 4.85° in pJIA, respectively. The angular deviations of the right and left foot PIP joints were 2.81° ± 11.25° and 2.5° ± 10° in oligoJIA, and 6.21° ± 11.53° and 6.07° ± 10.69° in pJIA, respectively. Angular deviations of the right and left hand PIP and distal interphalangeal (DIP) joints were observed only in pJIA. However, only right hand PIP joint deviation was noted in sJIA.

**Table 1:** Demographic data of the patients with JIA

Age	Frequency	Percent (%)
<5 years	05	12.5
5-10 years	27	67.5
11-15 years	06	15
>15 years	02	05
Sex		
Male	23	57.5
Female	17	42.5
Duration of JIA		
<12 months	23	57.5
12-36 months	15	37.5
>36 months	02	05
Any deformity		
Absent	11	27.5
Present	29	72.5
JIA categories		
pJIA	14	35
oJIA	16	40
SoJIA	05	12.5

**Table 2:** Angular deviation of the involved joints in degree

Joints	Oligo JIA (*n* = 16)	Prevalence (%)	pJIA (*n* = 14)	Prevalence (%)	sJIA (*n* = 5)	Prevalence (%)
Knee Right	2.00 ± 4.16	25	13.36 ± 17.03	57.14	3 ± 6.71	20
Knee Left	1.87 ± 5.12	18.75	12.5 ± 15.08	64.29	2.4 ± 5.37	20
Ankle Right	2.62 ± 5.06	25	5.43 ± 8.21	42.86	4.00 ± 8.94	20
Ankle Left	0.94 ± 2.72	12.5	5.29 ± 8.26	35.71	0	0
Wrist Right	1.25 ± 3.41	12.5	4.07 ± 8.93	21.43	2.4 ± 5.37	20
Wrist Left	0.94 ± 3.75	6.25	4.14 ± 9.36	21.43	2 ± 4.47	20
Elbow Right	0.094 ± 2.72	12.5	7.14 ± 13.26	28.57	0	0
Elbow Left	5.00 ± 2.00	6.25	5.64 ± 9.92	28.57	0	0
MTP Right	0.94 ± 3.75	6.25	2.50 ± 1.00	21.43	0	0
MTP Left	0.62 ± 2.50	6.25	2.43 ± 4.85	21.43	0	0
Foot PIP joint Right	2.81 ± 11.25	6.25	6.21 ± 11.53	35.71	0	0
Foot PIP joint Left	2.5 ± 10.00	6.25	6.07 ± 10.69	35.71	0	0
Hand PIP joint Right	0	0	16.93 ± 16.19	78.57	8 ± 10.95	40
Hand PIP joint Left	0	0	17.5 ± 18.53	78.57	8 ± 10.95	40
Hand DIP joint Right	0	0	1.79 ± 3.72	21.43	0	0
Hand DIP joint Left	0	0	1.79 ± 3.60	21.43		0

The data of angular deviation was represented as Mean ± SD. Involvement of joints in oJIA, pJIA, sJIA only represented. MTP: Metatarsophalangeal joints, PIP: Proximal interphalangeal, DIP: Distal interphalangeal.

## Discussion

JIA is a multifactorial inflammatory disease and heterogeneous group of disorders. It is characterized by arthritis of unknown etiology that manifests before the age of 16 years, and persists for at least six weeks, and causes long-term morbidity.^[9,10]^ Several factors, including the disease severity, genetic and environmental factors, and comorbidities play an important role in the treatment of JIA. Potential outcomes are remission, persistent joint involvement, joint damage and deformity, functional limitations and overall quality of life even with treatment.^[11,12]^ Despite awareness and various modern treatments including biologics, about 50% of children suffer from JIA. It can cause joint deformities and limited joint movements, leading to limitation in daily activities and social participation in their adulthood.^[13]^ Present study, showed that males outnumbered the females. Previous studies from the European and Latin American populations have shown that JIA is more prevalent in females.^[10]^ Previous studies, have reported the mean age at onset ranged between 4.1 and 10.3 years. A previous study from India reported that JIA was common from the age of 12 years onwards. In present study, the average age was (8.05 ± 3.2) years, which was lower than reported in the previous Indian study.

In the present study it was found that the most commonly involved joints were knee (40%), followed by hand (32.5%), ankle (30%), wrist & foot (17.5% each), elbow (12.5%) and cervical joint (7.5%). These findings are consistent with those of Sen *et al*. and Menon *et al*., who reported that knees and ankles were the most commonly affected joints.^[10,14]^ Although Naz S *et al*. reported that the hand and wrist joint were most commonly involved in their study, the present study found higher involvement of small joints of hand (32.5%), particularly in the pJIA categories.^[6]^ In this category hand PIP joint involvement is most common (78.57%) followed by knee joint (64.29%).^[14–16]^ Previously, a study on 105 patients found that articular damage was present in 48.6%, and extra-articular damage was 21.9% in children.^[3]^

Maximum joint involvement with deformities were observed in pJIA and had a positive correlation with the duration of disease.^[17,18]^ Previously, it was reported that pJIA is an aggressive and erosive subtype that inhibits the growth of the affected joints and can lead to growth retardation.^[9,19]^ However, there is no standardized method to quantify joint deformities using angular deviation. In the present study, knee was most affected major joint, particularly the left knee. The angular deviation (13.36° ± 17.03°) of the left knee was higher than the right knee (13.36° ± 17.03°), with a predominance of 64% and 57% respectively, in pJIA. Apart from major joints, right and left hand PIP were severally affected with a deviation of 16.93° ± 16.19° and 17.5° ± 18.53° respectively, and prevalence of 78.57%. In pJIA, monitoring angular deviation of the knee and hand PIP joints may be useful for monitoring the treatment response. In previous studies, it was observed that oligoJIA occurs in between 24%–60% of JIA patients. In the study, oligoJIA was observed as 40% and angular deviation of major and minor joints were less than pJIA. The children suffering from oligoJIA were more likely to respond to treatment than those with pJIA which may be co-related with lower angular deviation of the joints. The presentation of sJIA are variable compared to other JIA subtypes, with extra-articular features include fever, hepatosplenomegaly, rash and lymphadenopathy being more prominent than joint involvement.^[20,21]^ In this study, 8.9% of patients were diagnosed with sJIA. The results was higher than those reported in India (8%) and South Africa (7.7%).^[22]^ In this study, joint involvement and angular deviations (in degrees) were observed in the knee, right ankle, and hand PIP joints. These measurements can aid in monitoring and treatment progress in sJIA. Other potential confounding factors that could influence angular deviation and the improvement of angular deviation after treatment of JIA were not addressed in this study and should be considered in future research.

## Conclusion

JIA is associated with single or multiple deformities in around 70% of patients, and disease duration is significantly associated with the presence of deformity and the number of joints in pJIA. In connection, the angular deviation of major and minor joints was higher in pJIA, followed by oligoJIA and sJIA, compared to other subtypes. This is the first study from India to understand the angular deviation in degree in deformed joints in JIAs. The angular deviation may be a parameters that can be used to monitor disease progression of various JIA subtypes.
